# Supplementary material for: Are carbon emissions trading and green financial instruments synergistic? -Comprehensive quantitative research based on content analysis
Source: PLoS One. 2024 Mar 7;19(3):e0298601. doi: 10.1371/journal.pone.0298601 (PMC10919601; doi:10.1371/journal.pone.0298601)
Supplement: S1 Data — (ZIP) [file pone.0298601.s001.zip › Data/地级市Z_F工作报告.zip!/║╙─╧/║╫▒┌╩╨/└┤╫╘╣1⁄2╓┌║┼í╛┬φ┐╦╩2╛▌═°í┐_2008.docx]

各位代表：

　　现在，我代表市人民政府，向大会作政府工作报告，请予审议，并请各位政协委员提出意见

　　一、2007年工作回顾

　　2007年，市人民政府在省委、省政府和市委的正确领导下，团结带领全市人民，认真贯彻落实科学发展观，积极构建和谐社会，开拓进取，扎实工作，圆满完成了市八届人大四次会议确定的各项目标任务。

　　——总体经济实力加快提升。全市生产总值278亿元，增长18.2%。财政一般预算收入14亿元，增长31.8%；一般预算支出27.6亿元，增长33.2%。

　　——三次产业结构进一步优化。一、二、三产业增加值分别达到43亿元、173亿元、62亿元，增长5.1%、22.9%、14.7%，占生产总值的15.5%、62.2%和22.3%。二、三产业增加值占生产总值的比重提高0.6个百分点，对经济发展的贡献率达到95.5%。规模以上工业增加值139.7亿元，增长26.9%；实现利润25.8亿元，增长56.4%。旅游直接收入超过1亿元，增长22.5%。

　　——发展动力活力显著增强。全社会固定资产投资161.8亿元，增长48%，其中城镇固定资产投资增长45.8%。金融机构各项存款余额比年初增长8.6%，各项贷款余额增长22.2%，实现了贷款增量高出存款增量的可喜变化。社会消费品零售总额55亿元，增长18.1%。

　　——人民生活水平得到提高。城镇居民人均可支配收入10912元，增长20.1%；农民人均纯收入4827元，增长18.8%。在岗职工平均工资16741元，增长22.1%。

　　一年来取得的这些成就，标志着我市“加快建设新鹤壁、中原崛起争先锋”迈出了坚实步伐。2007年重点抓了以下工作：

　　(一)坚持大力发展循环经济，着力促进经济社会又好又快发展。循环经济取得新进展。总投资69亿元的42个重点循环经济项目已建成20个、在建20个，完成投资34亿元。3个循环经济示范园区建设积极推进，牟山工业循环经济园区、淇县畜牧业循环经济产业区被列为省循环经济试点园区。规划面积6.5平方公里的金山生态经济园区建设开始启动。指导督促16家循环经济试点企业完善试点方案并组织实施，鹤煤集团被列为省循环经济试点企业。在中国·欧盟循环经济论坛上交流了经验，被确定为国家循环经济标准化试点市。节能减排成效明显。严把项目准入关，加快电力、金属镁、建材等重点高耗能行业技术改造，开工建设了余热发电等13个节能技改项目，加快了产业结构优化升级。开展了重点耗能企业节能行动，引导重点耗能企业实行合同能源管理，国家、省、市重点节能降耗监控企业，全部按要求编制实施了能源审计报告和节能规划。既有建筑节能改造取得新成效，被确定为中国既有建筑节能改造项目第二批示范城市。大力推广新型墙体材料、地源热泵、太阳能等新产品新技术和可再生能源的开发利用，推进由单一建筑节能向城市节能发展，城区新建建筑节能覆盖率达到100%，市委市政府综合办公楼等3项工程被确定为国家可再生能源建筑应用示范项目。加大“禁实”工作力度，关闭实心黏土砖瓦窑厂70家，在全省率先实现市区、县城全部“禁实”。单位生产总值能耗预计下降4%以上。加快环保基础设施建设，4座污水处理厂全部竣工并投入运行，3座垃圾无害化处理场建成投用。积极推进卫河流域综合治理，全面完成了综合整治任务。全市化学需氧量、二氧化硫排放量分别下降3.9%和5.9%，超额完成省定目标。城乡环境质量进一步改善。河流出境断面水质平均达标率达到80%，城市饮用水源地水质达标率保持100%。市区环境空气质量优良天数达到337天，超过省定目标38天。造林绿化8.1万亩，超额完成省定目标任务，林业生态建设走在了全省前列。中办国办环保联合检查组、国务院“双高”行业检查组，对我市近年来的环保工作给予了较高评价。

　　(二)坚持强力推进招商引资和大项目建设，加快培育壮大优势特色产业。招商引资取得新进展。引进市外资金85.5亿元，增长12.8%；落地投资规模超亿元项目32个，比上年增加6个。实际利用境外直接投资5512万美元，超额完成省定目标。天海集团收购郑州雪城科技公司并在新加坡成功上市，实现了我市企业上市零的突破。大项目建设迈上新台阶。年初确定的64个重点项目完成投资56.2亿元，为年度计划的104.3%。投资45.2亿元的电厂三期、投资近10亿元的盘石头水库建成并发挥效益。采煤沉陷区综合治理工程全年完成投资2.1亿元，建成房屋34.6万平方米，安置居民3000户；棚户区改造工程完成投资3.2亿元，建成房屋40万平方米。投资25.6亿元的煤化工一期开工建设。投资36.6亿元的鹤辉高速公路项目前期工作进展顺利。投资70亿元的电厂四期签订投资协议。投资10亿元的水泥四期前期工作启动。石武客运专线、长(鹤)兖铁路项目前期工作积极推进。煤化工一期的开工、电厂四期投资协议的签订，标志着我市新一轮大项目建设拉开了序幕。产业结构调整优化步伐加快。在招商引资和大项目建设带动下，全市大企业数量越来越多、规模越来越大，规模以上工业企业由上年的294家增加到338家，其中超亿元企业由82家增加到118家。煤电、食品加工、汽车零部件、金属镁、新型建材等优势特色产业占规模以上工业增加值的比重达到74.6%，提高4.4个百分点。食品加工业增加值达到30.9亿元，增长50.7%。“永达”商标被认定为“中国驰名商标”，实现了我市“中国驰名商标”零的突破。

　　(三)坚持统筹城乡发展，积极推进城镇化和新农村建设。城镇化进程继续加快。城镇化率达到46%，提高1.8个百分点。新区建成区面积新增3平方公里、达到25.1平方公里。投资1.5亿元建设了一批基础设施、公用事业项目，城市功能进一步完善。规划许可建筑面积近300万平方米，居全省前列。城市规划精细化管理试点小区创建工作扎实开展，居住小区开发品位明显提高。城中村改造取得重要进展。老区加快产业结构优化升级，积极推进基础设施、公用事业建设，大力治理违法建筑、整治城市面貌，人居环境得到改善，发展活力进一步增强。加快推进新老区连接带开发，积极打造连接新老区的特色经济带和生态带，新型农村社区建设、生态绿化、高档住宅开发等稳步展开。两县县城加快道路、绿化等基础设施建设和住宅小区开发，努力提高建设管理水平，功能和面貌得到进一步改善。小城镇建设管理取得积极进展。“四城联创”工作扎实推进，促进了城市建设管理水平的提高和精神文明建设。新农村建设成效显著。基础设施、社会事业向农村延伸覆盖加快，农民生产生活条件进一步改善。投资1.5亿元建设农村公路620公里，改善了308个行政村的道路状况，24.7万农民从中受益。新增沼气用户1.7万户，35%的适宜建沼农户用上了沼气。解决了9.1万农村居民的安全饮水问题。发放“两免一补”资金5049万元，惠及农村学生18万人。提前三年完成了省定教育资源整合优化任务。在全市所有乡镇探索建立流动图书站，强化了农村中小学图书室功能。在全省率先完成了农村中学实验室改造。提前完成了农村中小学课桌凳更新改造，惠及学校359所。扶持建设了46个示范性农村文化大院。建设了80个集实用技术培训、致富信息服务、先进文化传播于一体的农村信息文化中心，在全国产生了较好反响。在全省率先实现新型农村合作医疗覆盖所有县区，参合率达到92.7%，居全省前列。村标准化卫生室覆盖面居全省首位，进一步方便了群众就医治病。制定完善了城乡一体化的具体措施，积极引导鼓励民营企业等各方面力量参与新农村建设，体制机制创新取得新进展。农业发展水平进一步提高。粮食总产106.7万吨、单产439公斤，分别增长5.9%和5.5%，均创历史新高；15亩超高产攻关田玉米亩产1064.8公斤，创世界夏玉米亩产最高纪录。被评为全国粮食生产先进市。畜牧业产值、加工业产值分别达到50.2亿元和66亿元，增长5.5%和29.4%，畜牧业产值占农业总产值的比重达到65%。成功承办了全国春季农业生产工作会议。

　　(四)坚持解决好人民群众最关心最直接最现实的利益问题，努力构建和谐社会。投入9.8亿元的十件实事全部落实，人民群众享受到更多发展成果。就业再就业工作成绩显著。投入4119万元支持就业再就业，新增城镇就业5.1万人，下岗失业人员再就业2.1万人，完成省定目标比例均保持全省首位。动态消除了“零就业家庭”。农村劳动力转移就业24.1万人，被联合国开发计划署确定为统筹城乡就业项目城市。以培训促创业、以创业带就业效果明显，被确定为全国创业实训项目试点城市。社会保障体系进一步完善。探索出台了农民工参加医疗保险办法，农民工参加医疗、工伤保险人数分别达到1.1万人和1.8万人。为9.1万名城乡困难群众发放低保金5698万元，实现了动态管理下的应保尽保。完善了城乡困难群众救助体系，强化了救助长效机制，提高了救助实效。社会事业加快发展。积极推进教育教学改革和管理创新，切实改善城乡学校办学条件，推进城乡教育均衡发展，提高了总体教育水平。投入1.4亿元，加强公共卫生和医疗服务体系建设，提高重大疾病防治能力，努力解决群众看病难、看病贵问题。文化事业和文化产业发展加快，成功举办了庆祝建市50周年大型文艺演出等系列文化活动。城乡体育设施建设取得新成效，“全民健身与奥运同行”活动受到国家体育总局表彰。提前八年在全国率先实现广播电视村村通。科技事业取得新进展，综合科技进步水平前移3个位次，进入全省先进行列。深化公交运营管理体制改革，优化公交线路布局，方便了市民出行。人口自然增长率4.46%。，继续稳定了低生育水平。民主法制和社会稳定工作扎实推进。自觉接受人大及其常委会的监督，支持政协参政议政，认真办理人大代表建议和政协提案214件。大力推进政务公开，提高了政府工作透明度。深入开展法制宣传教育和依法治市工作，公民法律素质和社会法制化管理水平进一步提高。社会主义荣辱观教育和精神文明创建活动深入开展。双拥共建活动扎实推进，军政军民团结不断加强。各类安全事故起数下降36.1%。刑事、治安案件大幅下降，现行命案侦破率连年保持全省第1位。认真做好信访工作，保持了全省先进位次，有效化解了社会矛盾和问题。被评为全省平安建设先进市。

　　(五)坚持加强政府自身建设，进一步提高工作质量和服务水平。扎实开展“讲正气、树新风”主题教育活动，认真学习贯彻党的十七大精神，政府系统广大干部职工特别是领导干部的精神状态更加饱满，工作作风明显转变，促进了各项工作开展。进一步优化工作流程，推进电子政务建设，完善重点工作台账制度，提高了工作效率和质量。严格政务督查、效能监察、工作纪律和问责制，提高了政府的执行力和公信力。有效发挥“12345”市长热线等便民渠道的作用，及时收集和解决群众反映的问题，加强政府与群众、政府与企业的沟通交流，取得了良好的社会效果。廉政建设和反腐败斗争取得新成效，政风行风继续好转。

　　各位代表！

　　2007年，我市经济社会又好又快发展，取得了令人鼓舞的好成绩：主要指标增速保持全省先进位次，经济发展实现新突破；社会事业全面推进，和谐社会建设迈出新步伐；不少工作成为全省乃至全国的亮点，知名度美誉度有了新提高。这些成就的取得，归功于省委、省政府和市委的正确领导，归功于市人大、市政协的大力支持，归功于全市人民的团结奋斗。在此，我代表市人民政府，向辛勤工作在全市各行各业的广大工人、农民、知识分子、干部、驻鹤部队指战员、武警官兵、公安民警以及社会各界人士致以崇高的敬意！向关心、支持我市现代化建设的同志们、朋友们表示衷心的感谢！

　　当前，我市经济正处于加快转型、加速发展、加力爬坡的关键阶段，经济社会发展中还存在不少困难和问题：经济结构不尽合理，高科技企业和大企业集团少，自主创新能力较弱，服务业发展较为缓慢；社会事业发展仍然存在薄弱环节，一些关系群众切身利益、影响社会和谐的问题亟待解决；个别政府工作人员的责任意识、大局观念、工作作风、服务水平与新形势新任务和广大人民群众的要求还存在差距。对此，我们一定高度重视，采取有效措施认真加以解决。

　　二、2008年政府工作总体要求和奋斗目标

　　2008年是全面贯彻落实党的十七大精神的第一年，是完成“十一五”规划目标任务的关键一年，也是我市谋求经济社会发展新突破的重要一年，做好全年的工作意义重大。通过近年来的不懈努力，我市经济社会发展实现了历史性跨越，生机和活力明显增强；今年，中央和省进一步加大对发展方式转变、民生改善、“三农”工作、环境保护、生态建设等支持力度。这为我市经济社会又好又快发展奠定了坚实基础、提供了良好机遇。省委、省政府对我市近年来的工作给予充分肯定，对我市未来的发展寄予更大希望，要求我们继续保持好势头、奋力推进新跨越，坚持科学发展，坚持干事创业，坚持团结和谐，在全省率先全面建成小康社会。这既是对我们的肯定和鼓舞，更是对我们的鞭策和期待。做好今年的工作，我们也面临着土地、资源、资金、环境容量等约束因素，面临着更大的竞争压力。我们必须深刻认识和把握新形势新要求，始终抓住科学发展不动摇，始终抓住改革创新不停步，始终抓住改善民生不放松，始终抓住维护稳定不懈怠，努力使重要领域、重点工作和经济发展水平继续走在全省前列，推动整体工作迈上新台阶，不辜负省委、省政府和全市人民的厚望。

　　今年政府工作的总体要求是：以党的十七大精神为指导，深入贯彻落实科学发展观，紧紧围绕在全省率先全面建成小康社会的目标要求，围绕壮大总体实力和转变经济发展方式、保障和改善民生，强力推进招商引资和大项目建设，不断深化循环经济发展，加快城乡一体化进程，着力构建和谐社会，切实加强生态文明建设，进一步加强政府自身建设，推动经济社会又好又快发展。

　　今年的主要目标是：生产总值增长15%；财政一般预算收入增长16%；规模以上工业增加值增长23%；全社会固定资产投资增长25%；社会消费品零售总额增长15%；利用市外直接投资超过90亿元；城镇居民人均可支配收入、农民人均纯收入分别增长13%和10%；新增城镇就业3万人以上，下岗失业人员再就业8100人以上；人口自然增长率控制在5%。以下；单位生产总值能耗降低5.5%；化学需氧量、二氧化硫排放量分别下降5.8%和6.5%。

　　三、强力推进招商引资和大项目建设，加快壮大经济规模

　　坚定不移地把招商引资和大项目建设作为经济工作的重中之重，拓宽路子，完善措施，强力推进，力争在壮大优势特色产业和支柱型企业上实现新突破。

　　继续加大招商引资力度。扩大融资渠道，搭建多元化融资平台，下大力气推进企业上市，争取1-2家企业上市。瞄准上市公司和国内500强、行业100强企业，积极招大引强，加快推进重点企业战略重组，引进投资规模超亿元项目30个。抓好高尔夫球具及精密机械加工等一批投资规模超亿元在建项目，促其早日投产见效。抓好金属镁及镁合金深加工等一批投资规模超亿元签约项目，争取早日开工建设。推进多晶硅等一批在谈项目，力争早日签约落地。切实加大工作力度，争取更多的政策和资金支持。

　　坚持不懈抓好大项目建设。围绕做大做强优势特色产业、强化基础能力建设，加快年初确定的重点项目建设，全年完成投资56亿元。加快煤化工一期、天海集团年产100万千米汽车电线等续建项目建设。鹤辉高速公路争取年内开工建设，煤化工二期、电厂四期、水泥四期、盐化工等项目争取早日开工。科学策划包装一批大项目，各县区至少谋划推进一个投资规模超10亿元的项目。做好石武客运专线、长(鹤)兖铁路、安新高速公路改扩建、南水北调中线工程、成品油管道、天然气管道等过境项目的协调服务，争取项目规划设计最大限度地满足我市发展需要，确保开工项目在我市顺利建设。

　　扶持重点企业做大做强。大力培养、引进企业经营管理人才，着力造就一支具有战略思维、创新精神和现代经营管理能力的企业家队伍。完善激励约束机制，引导支持重点企业进一步提高经营管理水平，积极创建国家级、省级企业技术中心，增强自主创新和核心竞争能力，努力打造知名品牌，加快发展成为我市支柱企业。全市35户重点企业年销售收入力争增加50亿元，达到230亿元。

　　积极发展服务业。加强金融生态环境建设，落实对金融机构的激励政策，努力实现金融和经济社会发展良性互动。市城市商业银行上半年挂牌营运，积极引进战略投资者加快自身发展，增强支持地方发展的能力。做好县区农信社改革后续工作，抓好淇县农信社组建农村合作银行试点。深入挖掘特色旅游资源，加大项目策划、包装和推介力度。继续抓好淇河生态保护和科学开发，启动盘石头水库景区建设，努力打造“诗经淇河·生态鹤壁”特色文化旅游品牌。积极发展面向农村的服务业，新增农村超市210家，力争农村超市行政村覆盖面超过80%。完善商业网点规划布局，推进物流项目建设，积极引进品牌商业项目，加快传统服务业改造升级。

　　努力营造更好的发展环境。引导督促各县区各部门进一步强化“环境就是生产力”的观念，明确责任，完善措施，创新工作，积极主动为重点项目、企业发展提供优质高效服务。落实和完善重点项目建设责任制，及时妥善协调好涉农涉企等关系。积极改善人才、融资、中介服务等环境，大力倡导尊重投资者、经营者、创业者的社会氛围，尽力满足项目建设和企业发展更高层次的需求。完善基础设施，强化服务功能，提高工业集中区发展水平，更好发挥项目承载、资源聚合、经济先导的作用。加强督查和效能监察，严肃查处不作为、乱作为等行为，努力营造投资者放心、经营者安心、创业者顺心的发展环境。

　　四、深化循环经济发展，加快转变经济发展方式

　　围绕建设国家循环经济示范市的目标，扩大规模，拓展领域，提升层次，打造典范，促进经济发展质量效益整体提高，实现经济效益、社会效益、生态效益有机统一。

　　切实抓好循环经济项目。总投资40亿元的20个续建项目力争全部投产，其中8个项目上半年建成投产。总投资8亿元的15个项目尽早开工建设。积极谋划推进一批延环补链项目，进一步完善产业链条。依托朝歌日光新能源公司、河南太行全利集团等企业，加快太阳能等清洁能源的开发利用，大力发展节能环保制造业。

　　加快建设示范企业示范园区。科学定位发展方向和目标，4月底前完成第一批示范企业、示范园区创建方案评审并组织实施。围绕循环型农业、循环型工业、生态经济一体化示范区的定位，加快建设金山生态经济园区。引导支持鹤煤集团、同力水泥、维恩克、绿佳等企业和牟山工业循环经济园区、淇县畜牧业循环经济产业区，按照国家循环经济评价指标体系加大创建力度，在资源消耗、综合利用和废物排放等方面达到国内先进水平，力争两年内建成在全国具有示范意义的企业和园区。

　　积极拓展循环型城市体系。在山城区、鹤山区开展再生资源回收利用体系建设试点，探索构建以城区回收站点为基础、集散市场为核心、加工利用为目的的再生资源回收利用体系。启动中水回用、城市生活垃圾分类回收和资源化利用等项目。在农村推广小型垃圾造肥、生产沼气装置，使农村垃圾就地减量。

　　不断提升循环经济发展层次。按照建设国家循环经济标准化试点市的要求，尽快制定实施煤电、金属镁、食品加工、新型建材等产业标准化体系，为全国循环经济发展提供依据、树立标杆。积极谋划举办循环经济高层论坛和新技术新产品展览会，广泛开展循环经济技术交流，加快引进推广先进适用技术和产品，提高循环经济的科技含量。引导支持县区结合自身实际推进相关工作，打造新的特色和亮点。

　　五、加快城镇化和新农村建设，推进城乡一体化发展

　　围绕建设全省城乡一体化示范市的目标，坚持以城乡一体化统筹城镇化和新农村建设，注重城镇化与新农村建设有机结合，注重政策和体制机制创新，加快构建城乡经济社会协调发展新格局。

　　继续加快城镇化进程。坚持以人为本的理念和建设宜居城市的定位，深入开展“四城联创”，加快城市建设步伐，进一步增强城市聚集承载能力，提升城市形象和品位。城镇化率达到48%，提高2个百分点。全方位推进新区建设。新建续建道路21公里，加快启动、建设体育中心等一批社会事业项目，加快建设淇河公园、市级商业中心、商务写字楼等项目，改造天赉渠主干渠、二支渠东段等河段，新增绿地42万平方米，进一步增强城市功能，扩大建成区面积。积极推进采煤沉陷区综合治理、棚户区改造、故县新村、浚县农场等小区建设，促进人口向新区聚集。全面启动新区城中村改造，郭小屯小区9月底前完成搬迁入住，崔庄小区年内启动搬迁入住。实现新区建成区公交线路基本覆盖。高品位规划设计石武客运专线鹤壁站及站前广场。把优化产业结构与推进老区改造结合起来，增强老区发展活力，积极吸引社会资金参与老区改造开发，继续推进基础设施建设、公用事业发展和棚户区、背街小巷改造，加强周边村庄改造与治理，进一步改善老区居住环境。科学规划引导新老区连接带开发，编制完成并尽快实施金山生态经济园区局部控制性详细规划，努力在打造连接新老区的特色经济带和生态带方面取得新进展。两县县城建设发展要进一步创新思路，壮大产业支撑，扩大规模、完善功能，增加现代气息，提高带动能力。因地制宜推进重点镇建设，进一步改善镇区面貌，增强人口吸纳能力。积极开展城市精品建筑、小区、街道、街区创建工作，进一步抓好县城小区物业、环境面貌等方面的精细化管理，提高城市建设管理水平。

　　扎实推进新农村建设。全面贯彻落实中央、省“三农”工作方针政策，促进农业稳定发展、农民持续增收、农村更加和谐。围绕建设全省现代农业示范市的目标，大力发展现代农业。加快培养有文化、懂技术、会经营的新型农民和农村实用人才。认真落实粮食直补等支农惠农政策，加强农民负担监管，保护和调动农民的生产积极性。用现代工业理念改造提升传统农业，加快推进农业工业化。新增市级以上龙头企业15家、农民专业合作组织30家，推动农业产业化扩大规模、提升档次。抓好畜牧业规模发展和粮食、畜产品精深加工，畜牧业产值增长17%以上、占农业总产值的比重达到66%，食品工业产值、增加值分别增长20%和15%以上。切实抓好粮食生产，加快发展规模种植，打造5个万亩以上优质小麦玉米高产攻关示范区，粮食总产稳定在100万吨以上。认真组织实施国家粮食丰产科技等专项工程，抓好现代农业科技示范基地建设。加快大中型先进农业机械的推广应用，全市综合农业机械化程度达到76%以上。改造中低产田、发展节水灌溉各4万亩。引导浚县农场加快建设现代农业综合示范区。打造农业信息“进村入户”平台，推进农村信息化。加快农村基础设施建设和社会事业发展。巩固扩大“村村通”公路建设成果，实施村道联网工程，建设农村公路150公里，提高农村公路、客运站、招呼站管理维护水平，切实方便农民出行。解决5万以上农村居民的安全饮水问题。农村沼气用户达到宜建农户的50%以上，建成沼气建设示范村10个。加大城乡教师交流力度，搞好农村中小学校舍维修改造，启动农村小学科学实验室改造，进一步改善农村办学条件。加大农村标准化卫生室建设管理力度，全市行政村基本建有标准化卫生室，更好地为农民服务。新建示范性农村文化大院30个、“农家书屋”50家，整合相关资源建设农村信息文化中心120个，完善农村基层信息文化服务功能。推进有线电视由城市向农村延伸。巩固扩大新农村建设试点成果。进一步扩大试点范围，试点村总数达到150个，促进市县乡三级试点村互动并进、协调发展。督促支持试点村加快发展经济、增加农民收入，抓好环境卫生综合整治，改善农村人居环境，改变农村整体面貌，努力建设农村新社区，增强示范带动作用。实现县区经济更大规模更高水平的发展。引导支持县区切实解放思想、创新思路、扩大开放，抓住重点，发挥优势，加压奋进，在全省全国大背景下加快壮大经济实力，加快推进工业化和城镇化，为我市在全省率先全面建成小康社会作出应有的贡献。

　　搞好城乡发展规划管理和体制机制创新。按照统筹城乡规划的要求，高标准修编城市总体规划，抓紧编制新区南部片区、老区拟改造地块等控制性详细规划，做到近期拟开发地区控制性详细规划全覆盖。编制新区与淇县县城连接带、京港澳高速公路浚县下道口周边区域控制规划，加强规划和土地利用管理。积极探索完善基础设施、改善公共服务、加强社会管理等统筹城乡发展的政策措施，着力在户籍管理、土地流转、社会保障等方面探索创新，进一步健全长效机制，促进城乡协调发展。

　　六、着力保障和改善民生，扎实推进和谐社会建设

　　注重发展成果的普惠性，努力使广大人民群众学有所教、劳有所得、病有所医、老有所养、住有所居。

　　坚持优先发展教育。全面推进素质教育。为农村义务教育阶段学生免费提供教科书，从秋季起免除城市义务教育阶段学生杂费。提高农村义务教育阶段中小学生人均公用经费标准、贫困家庭寄宿生生活费补助标准。积极解决农民工子女就学和城市部分中小学大班额问题。在全市农村乡镇政府所在地的小学、初中建设多媒体教室，市直学校30%以上的教室安装多媒体设备。认真落实职业学校学生助学机制，合理调整专业结构，培养更多的实用人才。积极推进高等教育发展，重视高等院校引进工作。大力发展民办教育。

　　做好就业和收入分配工作。全面落实积极就业政策，稳步扩大就业规模。实施创业带动就业战略，大力倡导、支持、推进全民创业，积极鼓励支持非公有制经济和劳动密集型产业发展。健全统筹城乡的就业服务体系和就业援助制度，筹建市级新型农民培训中心，完成农民工技能培训2.8万人以上、农村劳动力转移就业24.5万人以上。及时帮助“零就业家庭”解决就业困难，动态消除“零就业家庭”。进一步规范劳动关系，依法维护劳动者合法权益。建立工资支付保障机制，解决好拖欠职工和农民工工资问题。指导督促企业建立职工工资正常增长机制。全面落实最低工资保障制度，努力提高低收入者的收入水平。实施职工带薪休假制度。

　　大力发展医疗卫生事业。巩固完善新型农村合作医疗制度，为参合农民提高补助标准、降低医疗费用、建立健康档案，提前实现基本覆盖全市农村居民的目标。稳步推进医疗卫生体制改革。加快城市社区卫生服务体系建设，建成4家社区卫生服务中心(站)。加强医德医风建设，提高医疗服务水平。认真做好疾病防治工作。普及卫生科学知识，倡导文明健康科学的生活方式。

　　不断完善社会保障体系。加大社会保险费征缴力度，保证各项社会保险金按时足额发放。完善城镇企业职工基本养老保险制度，继续提高企业退休人员基本养老金水平。积极推进事业单位养老保险制度改革，探索建立农村养老保险制度。完善城镇职工基本医疗保险制度，启动城镇居民基本医疗保险工作。加快推进失地农民社会保障工作。进一步扩大农民工参加医疗、工伤保险覆盖面。提高城乡低保补差标准，实现应保尽保。提高农村五保供养标准，集中供养率达到40%以上。健全覆盖城乡、动态管理的长效救助机制。大力发展社会福利和慈善事业。新建或收购廉租住房1万平方米，扩大廉租补贴覆盖面。采煤沉陷区综合治理工程新开工5万平方米、竣工40万平方米以上，安置受损、动迁户3000户以上。全面完成棚户区改造任务。

　　扎实推进文化建设。加快构建市县乡村四级公共文化服务体系，保障人民群众的基本文化权益。进一步拓宽市文化中心服务功能，充分发挥流动图书网点、流动舞台的作用，将公共文化服务向县区、乡镇、社区和农村延伸。在全市公共图书馆建设文化信息资源共享工程支中心。推广农村电影数字化放映服务，全市60%的行政村每村每月放映一场公益性电影。发挥我市历史文化资源优势，积极策划推进文化产业项目，深化文化体制改革，打造在全省全国有一定影响力的品牌，推进文化强市建设。加强社会主义核心价值体系建设，用社会主义荣辱观引领社会风尚，大力弘扬爱国主义、集体主义、社会主义思想。加强社会公德、职业道德、家庭美德、个人品德建设，引导人们自觉履行法定义务、社会责任和家庭责任。做好青少年思想道德教育工作。加强网络监督管理。深化文明城市、文明行业和文明单位创建活动，广泛开展科教、文体、法律、卫生进社区和文化、科技、卫生“三下乡”活动，举办纪念改革开放30周年、迎奥运全民健身等活动。

　　切实加强社会管理。完善和加强城市社区功能，开展农村社区建设试点。做好第六届村委会换届工作。认真落实安全生产责任制，切实加强安全生产监管，坚决遏制重特大事故发生。强化食品药品安全监管，保障人民群众饮食用药安全。加强信访工作，有效预防、化解各类社会矛盾和问题。健全社会治安防控体系，依法防范和打击各类违法犯罪，深化法制宣传和依法治市工作，深入开展平安创建活动。稳定低生育水平，优化人口结构，提高人口素质。深入开展国防教育，支持驻鹤解放军和武警部队建设，抓好民兵预备役和人民防空工作，筹建民兵综合训练基地和武警支队教导队。认真落实优抚安置政策，积极开展双拥共建活动，巩固和发展军政军民团结。发挥工会、共青团、妇联、工商联等人民团体的桥梁纽带作用。继续做好民族、宗教、外事、对台、侨务、统计、气象、地震、档案、史志等工作。

　　下力气抓好承诺实事的办理。集中方方面面的力量，采取切实有效的措施，确保办好今年已向群众承诺的实事。

　　七、切实加强生态文明建设，增强可持续发展能力

　　围绕创建国家级生态市的目标要求，市、县(区)抓紧编制实施国家级生态市建设规划纲要，积极构建资源节约型、环境友好型的产业结构、增长方式和消费模式。

　　强化能源资源节约。加强目标管理，实行严格的问责制和“一票否决”制。继续推进重点企业节能降耗工作。推进余热余压利用等项目，有效降低高耗能行业能耗水平。严格执行节能环保准入标准，大力发展节能环保产业，坚决淘汰落后生产能力。建立完善节能产品、技术和合同能源管理等节能服务机制，提高能源利用效率。设立专项资金，支持节能技术研发和节能工程实施。严格执行建筑节能设计标准，积极推广新型墙材，启动3万-5万平方米既有居住建筑节能改造。探索推行供热分户计量、按用热量收费制度。严格耕地保护和土地管理，大力推进土地资源节约集约利用。加强节约能源资源宣传，大力倡导生态消费。

　　加强环境污染治理。完善污水处理配套管网，淇滨、山城污水处理厂加装脱氮装置，依法征收排污费和污水、垃圾处理费，确保污水处理厂、垃圾处理场稳定运营、达标排放。市区污水处理率达到85%以上，浚县、淇县污水处理率达到65%以上，全市城市生活垃圾无害化处理率达到70%以上。完成“小火电”、年生产能力2万吨以下再生纸企业关闭，以及万和发电公司烟气脱硫治理工程验收工作。重点治理化工助剂行业，完成山城区化工企业环境综合整治。抓好清洁生产审核工作。积极推进农村小康环保行动计划，对全市所有规模化畜禽养殖企业实施污染治理，控制和治理农村面源污染。加强饮用水源地保护和供水保障能力建设，饮用水源地水质达标率保持100%。继续抓好卫河流域水污染综合整治，实现河流出境断面水质稳定达标。全年市区空气质量优良天数达到330天以上。加强建设项目环境管理，严厉查处环境违法行为。抓好采煤沉陷区、资源枯竭矿井和矿山的环境修复与整治。

　　大力推进林业生态建设。完善林业生态建设规划，优化林业生态建设布局，突出抓好环城防护林、城郊森林、重点村镇绿化、生态廊道、平原林网、山丘经济林建设。完成造林绿化17.2万亩，全市森林覆盖率提高2.5个百分点。建立完善生态林管护长效机制。扎实推进集体林权制度改革，完成“四荒”拍卖承包5万亩。

　　八、加强政府自身建设，提高领导科学发展能力和服务水平

　　适应新形势新任务新要求，进一步加强政府自身建设，更好地履行职能，促进科学发展。

　　大力推进依法行政。严格执行人大及其常委会的决议、决定，自觉接受人大及其常委会的法律监督和工作监督。加强与政协的政治协商，自觉接受政协的民主监督。广泛听取各民主党派、工商联、无党派人士和社会各界的意见建议。严格按照法定权限和程序行使职权、履行职责。加强经济责任审计。建立完善行政执法责任制，真正做到严格执法、文明执法、公正执法。

　　不断提高服务水平。更加注重履行社会管理和公共服务职能，切实为社会、企业和人民群众提供优质便捷的服务。注重政务公开，增加政府工作透明度，保障人民群众的知情权、参与权、表达权、监督权。注重现代化服务手段和方式方法的运用，加强行政服务中心和电子政务建设。注重完善应急预案体系和管理体制，提高危机管理和抗风险能力。注重大胆探索、开拓创新，善于通过新思路新举措，解决改革发展中的新情况新问题，努力开创工作新局面。

　　切实抓好工作落实。健全绩效考核体系，把各方面工作引导到推动科学发展、促进社会和谐上来。落实和完善目标责任制，对省委、省政府和市委安排部署的工作，做到目标具体、责任明确、措施得力、奖惩到位。加强督导检查、跟踪问效，做到年初有安排、年中有检查、年末有考核，认认真真、切切实实把每一项工作抓好。

　　进一步转变作风。始终践行全心全意为人民服务的根本宗旨，更多地深入实际、深入群众，脚踏实地、埋头苦干，诚心诚意为广大人民群众办实事、办好事。始终做到讲大局、讲责任、讲纪律，继续营造和保持求真务实、干事创业、团结和谐的浓厚氛围。始终牢记“两个务必”，坦坦荡荡做人，干干净净做事，加强政府系统廉政和政风行风建设，坚决惩治和有效预防腐败，保持政府的良好形象，以实际行动赢得人民群众的信任和支持。

　　各位代表！

　　继续保持好势头、奋力推进新跨越，在全省率先全面建成小康社会，是人民寄予我们的殷切期望，是时代赋予我们的崇高使命。让我们在省委、省政府和市委的正确领导下，深入贯彻落实党的十七大精神，解放思想、改革创新，团结拼搏、开拓进取，为不断开创全市经济社会又好又快发展新局面，加快建设“富裕、文明、和谐、生态”新鹤壁而努力奋斗！
